# Transcriptomic identification and developmental mapping of *nrg3b* expression in zebrafish

**DOI:** 10.55730/1300-0152.2791

**Published:** 2025-12-29

**Authors:** Elham TARAHOMI, Nuray SÖĞÜNMEZ ERDOĞAN

**Affiliations:** 1 Department of Computational Applied Science and Engineering, School of Graduate Studies, Kadir Has University, İstanbul, Turkiye; 2 Department of Molecular Biology and Genetics, Faculty of Engineering and Natural Sciences, Kadir Has University, İstanbul, Turkiye

**Keywords:** Neuregulin 3b, zebrafish, in situ hybridization, transcriptomics, neurodevelopment, SCO-spondin

## Abstract

**Background/aim:**

Neuregulin 3 (NRG3) is an epidermal growth factor-like ligand that is involved in neuronal circuit formation in mammals. However, the expression profile of its zebrafish ortholog, *nrg3b*, remains poorly defined. The transcriptomic analyses of zebrafish *sco-spondin* mutants, which display alterations in their neurodevelopmental pathways, have indicated potential dysregulation of Nrg3b. Guided by these insights, this study aimed to characterize the temporal and spatial expression patterns of *nrg3b* in vivo in embryonic and adult zebrafish.

**Materials and methods:**

Publicly available RNA-seq data from heterozygous and maternal-zygotic zebrafish *sco-spondin* mutants at 5, 15, and 30 days postfertilization (dpf) were analyzed to evaluate differential expression, coexpression network positioning, and functional enrichment. Temporal expression of *nrg3b* was validated by quantitative reverse transcription polymerase chain reaction (RT-qPCR) in embryos at 1, 3, and 5 dpf. Spatial *nrg3b* localization at 5 dpf was validated by whole-mount in situ hybridization (WISH), alongside neuronal subtype specificity using GABAergic and glutamatergic markers. Adult brain expression was further evaluated via ISH on telencephalon sections.

**Results:**

Developmental stage was the main driver of early transcriptomic variation, with minor genotype-specific differences detected at 5 dpf. Network analysis positioned *nrg3b* as a hub gene within a neuronal function associated module. RT-qPCR showed a significant increase in *nrg3b* expression on days 3 and 5 compared to day 1. WISH revealed strong *nrg3b* expression in anterior brain regions and the spinal cord at 5 dpf, with greater overlap observed with the glutamatergic marker compared to the GABAergic one. In adults, ISH suggested the expression of *nrg3b* in telencephalic nuclei, particularly in dorsomedial and ventral regions.

**Conclusion:**

*nrg3b* exhibits stage-dependent upregulation and preferential enrichment in excitatory neuronal regions during development, with sustained expression in adult telencephalon. These findings suggest a potential role for Nrg3b in synaptic organization and provide a foundation for future studies on the Nrg3b–ErbB4 signaling axis in zebrafish neurodevelopment.

## 1. Introduction

Neurodevelopment is a dynamic process that involves the proliferation of neural progenitors, neuronal differentiation, migration, and the formation of functional neural circuits within the nervous system (Belmonte-Mateos and Pujades, 2022; Scuderi et al., 2025). These events require the coordination of extracellular signaling cues (Guan and Rao, 2003), intracellular transcriptional programs (Sanders et al., 2022), and cell–cell communication mechanisms that guide neural patterning and the formation of neural circuits (Su et al., 2024). Disruptions in early developmental pathways can result in long-lasting consequences for neural connectivity (Geschwind and Rakic, 2013), motor function (McLeod et al., 2014) and susceptibility to neurological disorders (Turker et al., 2023). Among extracellular regulators, cerebrospinal fluid (CSF) flow and matrix-associated proteins secreted into the ventricular system play essential roles in shaping neuronal growth and axonal guidance (Li et al., 2024).

Subcommissural organ (SCO)-spondin (Sspo) is a large multidomain glycoprotein in zebrafish, secreted by the subcommissural organ in the brain and is a structural component of the Reissner fiber (Muñoz et al., 2019; Sepúlveda et al., 2021). Mutations in *sspo* have been associated with spine deformities and scoliosis phenotypes in zebrafish (Rose et al., 2020), and the gene is also expressed in the earlier developmental stages (Troutwine et al., 2020). RNA sequencing of heterozygous and homozygous *sspo* zebrafish has revealed widespread transcriptional changes (Rose et al., 2020); however, the functional consequences of its neural or neuromuscular expression remain poorly characterized. The epidermal growth factor (EGF)-like domains in Sspo (Sepúlveda et al., 2021) situate it within a broader molecular context where its EGF-like motifs also define signaling proteins such as neuregulins (NRGs), a family with established roles in neural and neuromuscular development (Cantaut-Belarif et al., 2018) through their ability to function as soluble or membrane-anchored ligands and with broader signaling versatility (Falls, 2003).

The NRG family (NRG1–4) comprises EGF-like ligands that bind to ErbB receptor tyrosine kinases (Cespedes et al., 2018). Through ErbB activation, NRGs regulate processes that include cell proliferation, survival, and differentiation (Esper et al., 2006), synaptic development, and activity-dependent plasticity (Wang et al., 2017). Within the NRG family, NRG1 has been extensively studied and is essential for Schwann cell survival and myelination (Fledrich et al., 2014) and for the formation and maintenance of the neuromuscular junction (Lee et al., 2016). NRG2 and NRG4 have narrower profiles, with roles described in neuronal and epithelial contexts (Howard et al., 2021). NRG3 differs structurally from NRG1/2, lacking the Ig-like domain and functions primarily as a transmembrane ligand that signals through ErbB4 receptors (Vullhorst et al., 2017). Functions of NRG3–ErbB4 signaling include contributions to interneuron migration (Li et al., 2012), regulation of the excitatory–inhibitory balance (Müller et al., 2018) and synaptic stabilization (Zhou et al., 2018). However, the developmental expression and function of *nrg3* orthologs in zebrafish have not been fully characterized.

**Figure 1:**
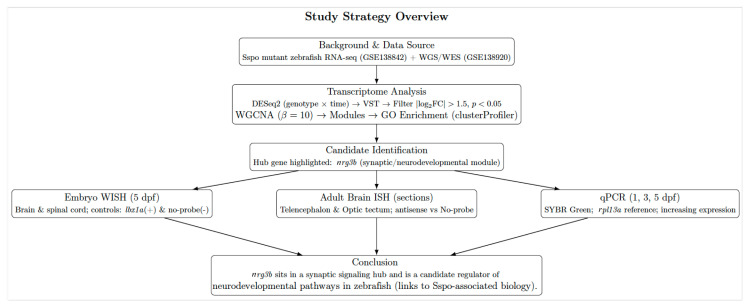
Graphical overview of the study strategy from dataset selection to *nrg3b* expression profile identification and its experimental validation.

This study focused on the zebrafish ortholog of *NRG3*, *nrg3b*, which was identified from the transcriptomic analyses of *sspo* mutant backgrounds as a candidate regulator of neurodevelopment. This work provides the first bioinformatics-informed experimental characterization of *nrg3b* expression in zebrafish and defines its temporal and spatial expression during early development, establishing a framework to explore how *sspo*-related transcriptional changes intersect with NRG signaling in neural and neuromuscular systems (Figure 1).

## 2. Materials and methods

### 2.1. Data source

RNA-Seq of a *sspo* transheterozygous (*sspo_hsc105/stl297_*) and *sspo* hypomorphic mutant (*sspo_stl297/stl297_*) zebrafish at different ages [5, 15, and 30 days postfertilization (dpf)] were obtained from the open-source National Center for Biotechnology Information Gene Expression Omnibus database (Troutwine et al., 2020; NCBI GEO ID: GSE138842). Throughout the manuscript, transheterozygous mutants refer to *sspo_hsc105/stl297_* animals, which are heterozygous at the zygotic level and retain maternal Sspo contribution, whereas hypomorphic mutants refer to *sspo_stl297/stl297_* animals, which are maternal-zygotic mutants lacking maternal Sspo contribution and carrying two hypomorphic alleles. Accordingly, the terms heterozygous and maternal-zygotic used in this study correspond to the transheterozygous (*sspo_hsc105/stl297_*) and hypomorphic (*sspo_stl297/stl297_*) mutant groups in the original dataset, respectively. Sample grouping by condition, timepoint, and replicate is summarized in the Table, where for each genotype and developmental stage, three independent biological replicates were analyzed.

### 2.2. Identification of differentially expressed genes (DEGs)

Differential gene expression analysis was performed using the DESeq2 package (v1.42.0) (Love et al., 2014). The design formula included both genomic status (maternal-zygotic vs. heterozygous) and timepoint (5, 15, and 30 dpf) as additive factors (design = ~ genotype + timepoint), allowing genotype-dependent transcriptional effects to be assessed while accounting for the dominant influence of developmental progression. Genes were filtered using the criteria of baseMean > 0, absolute log_2_ fold change (|log_2_FC|) > 1.5, and adjusted p < 0.05. Genes with NA adjusted p-values, typically arising from low base mean expression and independent filtering in DESeq2, were excluded from the DEG list (Love et al., 2014).

### 2.3. Enrichment of biological functions

Gene Ontology enrichment was performed using the clusterProfiler software package in R (Chen et al., 2013). Biological process ontology enrichment was performed, and terms with a Benjamini–Hochberg adjusted p-value ≤ 0.05 were considered significant.

### 2.4. Weighted gene coexpression network analysis (WGCNA)

The soft-thresholding power (β) was selected using the pickSoftThreshold function to approximate the scale-free topology; β = 10 was chosen based on the fit indices. The adjacency matrix was calculated and converted to a topological overlap matrix (TOM). TOM-based dissimilarity was used for clustering, and modules were identified using dynamic tree cut (module size_min_=30) and merged by eigengene correlation. Degree centrality indices were calculated and plotted using igraph (Csardi, 2013). Among genes within genotype-associated modules, *nrg3b* ranked among the most central nodes based on degree centrality, suggesting a potential regulatory relevance within the network. Therefore, *nrg3b* was prioritized for subsequent analyses.

### 2.5. Zebrafish maintenance and husbandry

Adult zebrafish (*Danio rerio*; ≥ 6 months of age) of the Tupfel Long Fin laboratory strain, as well as an in-house strain derived from a local pet store, were used for the experiments. The fish were bred, grown, and maintained at the Boğaziçi University Life Sciences and Technologies Center (İstanbul, Türkiye), which is Association for Assessment and Accreditation of Laboratory Animal Care International (AAALAC)-accredited for laboratory animal care. Maintenance conditions included a temperature of 27 °C with a light/dark photoperiod of 14:10 h in professional aquatic systems (Aquatic Habitats, Apopka, FL, USA). The fish were fed flake food (TetraMin; Tetra GmbH, Melle, Lower Saxony, Germany) twice daily and live brine shrimp larvae (*Artemia sp*.; Fides Artemia Co., İstanbul, Türkiye) once daily.

All experiments and procedures were performed in accordance with the guidelines and laws related to the use of animals in biological research, including the National Animal Protection Law (Turkish Law No. 5199, published on 24.06.2004 and amended on 30.07.2024); Directive 2010/63/EU of the European Parliament and of the Council of September 22, 2010; and the Guide for the Care and Use of Laboratory Animals (National Research Council, 2011) published by the AAALAC.

The animals were anesthetized and euthanized using tricaine (MS-222; ethyl 3-aminobenzoate methanesulfonate; Sigma-Aldrich Chemical Co., St. Louis, MO, USA; Cat. No. A5040) at a final concentration of approximately 170 mg/L prepared in clean system water prior to tissue collection.

### 2.6. Brain tissue preparation

The brains were dissected under a stereomicroscope and fixed overnight at 4 °C in 4% paraformaldehyde (PFA) in phosphate-buffered saline (PBS) (pH 7.4). Subsequently, the tissues were cryoprotected in 30% sucrose (w/v) in PBS at 4 °C until they sank. The brains were then embedded in optimal cutting temperature compound (Tissue-Tek, Sakura Finetek, Japan), frozen at −80 °C, and sectioned coronally at 12 μm using a Leica CM1520 cryostat (Leica Microsystems, Wetzlar, Germany). Sections were mounted on Superfrost Plus slides (Thermo Fisher Scientific Inc., Waltham, MA, USA), dried at 65 °C for ~2 h, and stored at −80 °C until further use.

### 2.7. RNA probe synthesis

Digoxigenin (DIG)-labeled RNA probes were synthesized according to standard in situ hybridization (ISH) procedures (Thisse and Thisse, 2008). Total RNA was extracted and reverse-transcribed into cDNA using oligo(dT) primers. Antisense probe templates were generated via polymerase chain reaction (PCR) using gene-specific primers in which the reverse primer carried a T7 promoter sequence at the 5′ end. *nrg3b* was used as the primary gene of interest. As a positive control, the *ladybird homeobox 1a* (*lbx1a*) gene, whose expression is well characterized in zebrafish (ZFIN: ZDB-GENE-040724-40; Howe et al., 2021), was also included. To assess neuronal subtype specificity, additional probes were synthesized for *slc17a6a* (Vglut2a; glutamatergic marker) and *gad1b* (glutamic acid decarboxylase 1b; GABAergic marker). PCR products were confirmed on a 1% agarose gel, purified using a PCR purification kit (New England Biolabs, Inc., Ipswich, MA, USA), and used as templates for in vitro transcription with T7 RNA polymerase and DIG-labeled nucleotide mix (Roche Group, Basel, Switzerland) at 37 °C for 2 h. Following DNase I treatment, the probes were purified, and their integrity and concentration were assessed via agarose gel electrophoresis and ultraviolet (UV) spectrophotometry (NABI UV-Vis; North American Biomedical Institute LLC, St. Petersburg, FL, USA). All probes were stored at −80 °C until further use. Primer sequences are listed in Table S1.

### 2.8. Whole-mount ISH (WISH)

WISH was performed following standard protocols (Thisse and Thisse, 2008) with minor modifications. Embryos were fixed in 4% PFA overnight at 4 °C, dehydrated in 100% methanol, and stored at −20 °C. Following rehydration through a methanol/PBT series, samples were bleached (3% H_2_O_2_/0.5% KOH), digested with 10 μg/mL proteinase K (30 min for 5 dpf embryos), and acetylated in 0.3% acetic anhydride/1% triethanolamine. After re-fixation in 4% PFA, embryos were prehybridized and hybridized overnight at 70 °C with DIG-labeled antisense probes. Posthybridization washes were carried out at 70 °C using graded SSC/formamide solutions, followed by PBT washes. Samples were blocked (5% sheep serum, 2 mg/mL BSA, 1% DMSO in PBT) and incubated overnight at 4 °C with anti-DIG-AP antibody (1:7500, Roche). Signal was developed using NBT/BCIP in NTMT buffer and monitored until optimal staining was achieved. Embryos were re-fixed in 4% PFA and cleared in a glycerol/PBT gradient prior to imaging.

### 2.9. Sectioned brain in situ hybridization (ISH)

Chromogenic ISH was performed on cryosectioned adult zebrafish brain. Sections were circled with a PAP pen, washed in 1X PBS, and fixed in 4% PFA. After permeabilization with 10 μg/mL proteinase K, samples were refixed, washed in PBST, treated with 0.2 M HCl, and acetylated. Sections were prehybridized for 15 min and hybridized overnight at 65 °C with 1 ng/μL DIG-labeled RNA probes in hybridization buffer (50% formamide, 5X SSC, 0.1% Tween-20, heparin, yeast tRNA, 5 mM citric acid). Posthybridization washes were performed in graded SSC/Tween-20 at 65 °C. Samples were blocked in 0.2% BSA/PBST and incubated overnight at 4 °C with anti-DIG-AP antibody (1:700, Roche). Signal was developed using NBT/BCIP in NTMT buffer and monitored for 2–16 h. Slides were washed in PBS and stored for imaging.

### 2.10. Quantitative real-time PCR

Total RNA was extracted from zebrafish embryos at 1, 3, and 5 dpf (three biological replicates per stage, 15 pooled embryos per replicate) using Trigent reagent (Biomatik; AGPC-based). RNA purity and integrity were verified spectrophotometrically and by agarose gel electrophoresis, respectively. Ultra cDNA Synthesis Kit (PCR Biosystems, UK) was used for cDNA synthesis. No-RT controls were used to assess gDNA contamination.

Quantitative reverse transcription PCR (RT-qPCR) was performed on each biological replicate in three technical replicates. Reactions were conducted on a Roche LightCycler using Luna Universal qPCR Master Mix (SYBR Green, New England Biolabs, USA) in a total volume of 10 μL, containing 1X mix, 200–400 nM of each primer, and ~10 ng cDNA. The cycling conditions were as follows: initial denaturation at 95 °C for 2–3 min, followed by 40 cycles at 95 °C for 15 s and 60 °C for 30 s, with data acquisition in the annealing/extension step. A melting curve (65–95 °C) was included to verify amplification specificity. Ribosomal protein L13a (*rpl13a*) was used as the reference gene. Relative expression levels at 3 and 5 dpf were calculated using 1 dpf samples as the calibrator (Livak and Schmittgen, 2001). Primer sequences are listed in Table S2.

### 2.11. Data analysis

Data were processed using GraphPad Prism 9 software, and statistical analysis was performed using one-way ANOVA followed by Dunnett’s post hoc test, comparing each developmental stage to day 1 as the calibrator. Statistical significance was defined as *p < 0.05, **p < 0.01, ***p < 0.001.

## 3. Results

### 3.1. Transcriptomic prioritization of *nrg3b*

**Figure 2:**
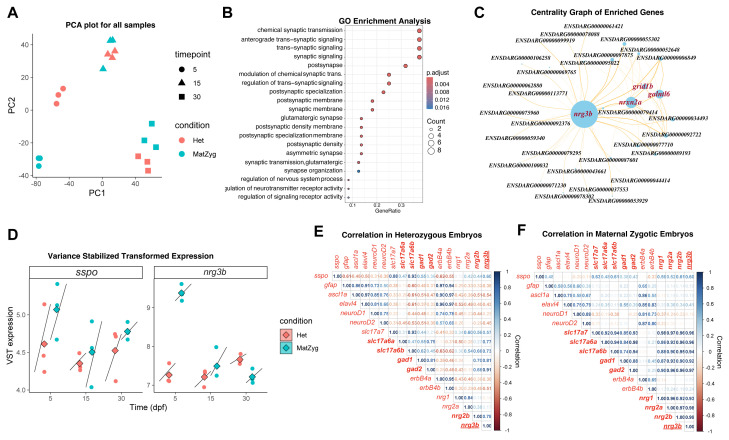
Transcriptomic prioritization of *nrg3b* in zebrafish. A, PCA of the variance-stabilized RNA-seq data showing clustering of samples mainly based on developmental stages (5, 15, and 30 dpf). Het: *Sspo* heterozygous mutant, MatZyg: *Sspo* maternal-zygotic mutant. B, Gene ontology enrichment analysis. C, Coexpression analysis showing high centrality of *nrg3b* and its connectivity to synaptic genes. D, Variance stabilizing transformation expression pathways for *sspo* and *nrg3b* developmental stages in heterozygous (brick) and maternal-zygotic (cyan) embryos. E, Correlation matrix of synaptic and neuronal markers in heterozygous embryos. F, Correlation matrix of synaptic and neuronal markers in maternal-zygotic embryos. Anticorrelation (red), positive correlation (blue).

Sspo, a Reissner fiber glycoprotein with EGF-like domains (Muñoz et al., 2019; Sepúlveda et al., 2021), is linked to zebrafish spinal defects and widespread transcriptional changes (Rose et al., 2020; Troutwine et al., 2020), suggesting potential neurodevelopmental signaling roles analogous to NRGs. Our bulk RNA-seq analysis of heterozygous and maternal-zygotic *sspo* zebrafish mutants revealed stage-dependent expression dynamics and subtle genotype-specific effects, providing a framework to identify candidate regulators of neurodevelopment. First, principal component analysis (PCA) was performed on the RNA-seq data to reduce dimensionality and visualize the overall patterns of gene expression in the samples as shown in Figure 2A. PC1 and PC2 explained most of the variance, with the samples clearly clustering by developmental stage (5, 15, and 30 dpf), indicating that development was the dominant factor influencing transcriptional profiles. At 15 and 30 dpf, heterozygous and maternal-zygotic samples largely overlapped, showing minimal genotypic variation, whereas at 5 dpf, a modest separation between genotypes suggested early, transient genetic effects.

To further elucidate the molecular context of these early transcriptional differences, gene ontology enrichment analysis (Ashburner et al., 2000), was performed on the dataset (Figure 2B). The analysis showed significant overrepresentation (FDR < 0.01) of biological processes, such as synapse organization, glutamatergic synaptic transmission, postsynaptic density, postsynaptic membrane, regulation of trans-synaptic signaling, and modulation of chemical synaptic transmission. The enrichment of these functionally related terms suggested that early transcriptional changes may converge on pathways governing synapse formation and maintenance. The prominence of glutamatergic signaling and postsynaptic specialization suggests a connection of this module to activity-dependent neural connections and plasticity.

Coexpression network analysis (Figure 2C) revealed a module that is highly enriched in genes associated with synaptic signaling and neuronal development. Within this network, *nrg3b* emerged as the principal hub gene based on degree centrality, indicating extensive coexpression connectivity. Interestingly, *nrg3b* was coexpressed with *nrxn2a*, *galntl6*, and *grid1b*, genes known to play essential roles in synapse formation, organization, and posttranslational modification of synaptic proteins (Ju et al., 2020; Gomez et al., 2021; Ung et al., 2024). These interactions identify *nrg3b* as a transcriptionally associated component in synaptic gene networks and suggest it as a suitable candidate for further investigation in the context of Sspo-related neurodevelopmental mechanisms. Moreover, the dynamic expression pattern of *sspo* (Figure 2D) showed an increase at 5 dpf, a decrease at 15 dpf, and a modest increase again at 30 dpf. This variation was observed in both heterozygous and maternal-zygotic genotypes, indicating that developmental stage, rather than genetic status, primarily accounts for these changes. In contrast, *nrg3b* expression was higher in maternal-zygotic embryos than in heterozygotes at 5 dpf, but this difference diminished at 15 and 30 dpf. These observations suggest that *nrg3b* expression is developmentally regulated, where 5 dpf is the first stage at which transcriptional differences between genotypes become apparent.

To assess how *nrg3b* relates to neuronal signaling programs, a curated gene set was analyzed, including *sspo*, *nrg1/2*, *erbb4a/b*, *ascl1a*, *elavl4*, *neurod1/2*, *slc17a7*, *slc17a6a/b*, *gad1*, and *gad2*, representing regulators of neuronal differentiation and synaptic activity (Grone and Maruska, 2016; Tutukova et al., 2021). *Nrgs* and *erbb4* paralogs were examined to explore potential ligand–receptor relationships, while *Ascl1a*, *Elavl4*, and *Neurod* markers were used to indicate progenitor activation and neuronal maturation (Tutukova et al., 2021). The *slc17a* genes mark glutamatergic neurons, and *gad1/2* mark GABAergic neurons (Higashijima et al., 2004; Grone and Maruska, 2016), consistent with findings that *Nrg3* is expressed in both neuronal subtypes during mammalian development (Müller et al., 2018).

Correlation analysis revealed distinct *nrg3b* coexpression patterns between heterozygous (Figure 2E) and maternal-zygotic (Figure 2F) *sspo* mutants. In heterozygotes, *nrg3b* correlated more strongly with inhibitory markers (*gad1*, r = 0.81; *gad2*, r = 0.91) than excitatory ones (*slc17a6a*, r = 0.77; *slc17a6b*, r = 0.73), indicating preferential transcriptional association with inhibitory circuits in partial Sspo deficiency. In maternal-zygotic embryos, correlations were uniformly stronger across both inhibitory (*gad1*, r = 0.92; *gad2*, r = 0.97) and excitatory markers (*slc17a6a*, r = 0.92; *slc17a6b*, r = 0.94; *slc17a7*, r = 0.96), suggesting broader coordinated expression across neuronal marker genes upon Sspo loss. Within the NRG family, *nrg3b* correlated mainly with *nrg2b* (r = 0.78) in heterozygotes, but showed strong coexpression with *nrg2b* (r = 0.99), *nrg1* (r = 0.93), and *nrg2a* (r = 0.98) in maternal-zygotic mutants. Correlation with *erbb4a* and *erbb4b* shifted from moderate negative (r = −0.38; −0.51) in heterozygotes to weakly positive (r = 0.09; 0.24) in maternal-zygotic embryos. While these changes indicate a reorganization of transcriptional coupling, they do not imply direct functional regulation or ligand–receptor interaction between Nrg3b and ErbB4 members. Instead, the observed shifts may reflect broader network-level transcriptional remodeling associated with Sspo deficiency, or alternatively, independent regulatory mechanisms governing ErbB receptor expression that are not directly linked to *nrg3b* activity.

### 3.2. Spatiotemporal profiling of *nrg3b* expression during zebrafish development

**Figure 3:**
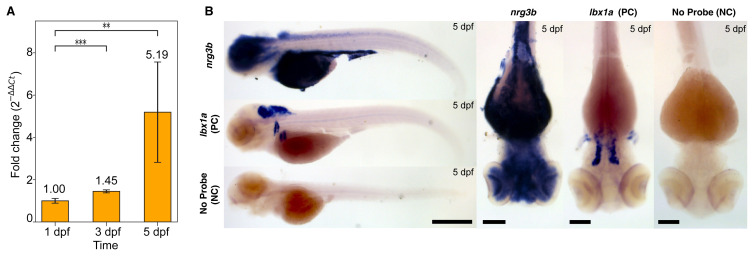
Validation of *nrg3b* expression at 5 dpf. A, RT-qPCR validation of *nrg3b* expression at 1, 3, and 5 dpf. Expression was normalized to day 1 using *rpl13a* as the reference gene. Data represent the mean fold-change ± SEM (n = 3 biological replicates × 3 technical replicates per time point). One-way ANOVA showed a significant effect of the developmental stage (F_(2,24)_=9.99, p < 0.001). Post hoc Dunnett’s test indicated significant upregulation on days 3 and 5 vs. day 1. B, Detection of *nrg3b* expression in embryos. WISH in 5 dpf zebrafish embryos using *nrg3b*, *lbx1a* (positive control (PC)), and no-probe (negative control (NC)). Lateral views (left) show whole-body expression; dorsal views (right) show the head region. Scale bars: 500 μm for the whole body, 100 μm for the dorsal views.

To further validate our bioinformatic predictions that 5 dpf represents a critical transcriptional window, *nrg3b* expression was quantified during early developmental stages using quantitative reverse transcription PCR (RT-qPCR). This approach was informed by two observations: first, the bioinformatics analysis revealed the first genotype-dependent divergence at 5 dpf; second, zebrafish single-cell transcriptomic data, publicly available in the DanioCell database^1^, also reports a peak *nrg3b* expression at 5 dpf (Figure S). To test this experimentally, transcript levels on days 1, 3, and 5 dpf, were measured (Figure 3A).

Expression levels were normalized to day 1 as a calibrator. One-way ANOVA revealed a significant overall effect of developmental stage on *nrg3b* expression (F=9.99, p < 0.001). Post hoc Dunnett’s tests confirmed that *nrg3b* was significantly upregulated on day 3 (mean fold change of ~1.45-fold, p < 0.05 vs. D1) and further increased on day 5 (mean fold change of ~5.19-fold, p < 0.05 vs. D1) (Figure 3A). At 5 dpf, *nrg3b* expression reached its highest mean level but also showed greater variability among biological replicates, resulting in a larger standard deviation (Dunnett’s test: **p < 0.01) compared to 3 dpf (Dunnett’s test: ***p < 0.001). These results validate a progressive early transcriptional induction of *nrg3b* between days 1 and 5 dpf. The marked rise on day 5 is consistent with the computational analysis and independent single-cell atlas data, supporting this stage as the peak of *nrg3b* activity.

To determine the spatial expression pattern of *nrg3b* at 5 dpf, WISH was performed on zebrafish embryos. Strong and central nervous system-specific expression of *nrg3b* was observed in the embryonic head (Figure 3B). Lateral views showed intense staining in the forebrain and midbrain that extended posteriorly along the spinal cord, indicating widespread neural expression. The signal was strong in the anterior neural tube, consistent with its roles in early brain development.

*lbx1a*, a regulator of neuronal pattern formation in the hindbrain and spinal cord (Gross et al., 2002), was used as a positive control and showed expression in bilateral clusters in the hindbrain and dorsal spinal cord, confirming protocol accuracy and regional specificity. In contrast, negative control samples (without probe) showed no detectable signal, confirming the specificity of the hybridization signal. Dorsal views also highlighted *nrg3b* expression in the brain, particularly in the forebrain region, while *lbx1a* expression remained restricted to segmental clusters in the hindbrain (Figure 3B).

### 3.3. Embryonic expression landscape of *nrg3b* relative to *gad1b* and *slc17a6a*

**Figure 4:**
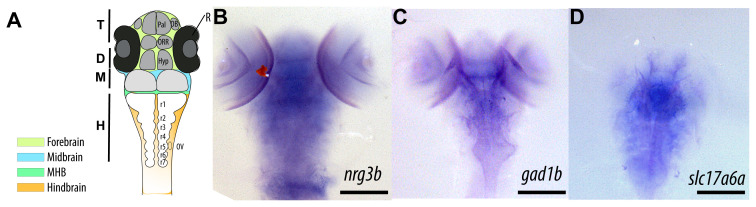
Comparative analysis of *nrg3b* expression with glutamatergic and GABAergic markers in the 5 dpf zebrafish. A, A schematic overview of the coronal zebrafish embryonic brain. The illustration is not drawn to scale. Distinct color codes highlight the principal brain regions: the forebrain (yellow), midbrain (blue), midbrain–hindbrain boundary (green), and hindbrain (orange). The forebrain further differentiates into the telencephalon (T, darker gray) and diencephalon (D, lighter gray), the latter encompassing the hypothalamic domain. T, telencephalon; D, diencephalon; M, midbrain; MHB, midbrain–hindbrain boundary; H, hindbrain; Hyp, hypothalamus; OB, olfactory bulb; ORR, optic recess region; OV, otic vesicle; Pal, pallium; R, retina; r1–r7, rhombomeres 1–7. Scale bars: 100 μm. Adapted conceptually from (Vaz et al. 2019). B, ISH for *nrg3b* at 5 dpf (dorsal view) shows a strong signal in the anterior forebrain and dorsal midbrain, extending slightly along the neural tube. A red signal detected in the retinal area corresponds to a staining artifact. C, ISH for *gad1b* (a marker of GABAergic neurons) shows a widespread expression pattern in the ventral forebrain, midbrain, and hindbrain, with the highest staining intensity in the optic tectum and ventral regions. D, ISH for *slc17a6a* (a marker of glutamatergic neurons) highlights areas of the forebrain and dorsal midbrain that partially overlap with the expression pattern of *nrg3b*. Scale bars: 100 μm.

Previous studies on rodents have shown that *Nrg3* is expressed in both GABAergic and glutamatergic neurons, with increasing levels during postnatal development and localization in synaptic compartments (Müller et al., 2018; Rahman et al., 2019; Ahmad et al., 2022). However, comparable data are lacking in zebrafish. To qualitatively assess whether *nrg3b*-expressing neurons are associated with excitatory or inhibitory domains in this species, WISH was performed using *gad1b* (a marker of GABAergic neurons) and *slc17a6a* (a marker of glutamatergic neurons) probes at 5 dpf (Figure 4).

The zebrafish embryonic brain is organized into distinct morphological domains that give rise to the major regions of the mature brain (Figure 4A). The forebrain occupies the anterior region and subdivides into the telencephalon and the diencephalon, which includes the hypothalamic territory. Posterior to the forebrain lie the midbrain and the midbrain–hindbrain boundary, followed by the hindbrain composed of segmented rhombomeres (r1–r7), establishing the structural framework for subsequent neuronal differentiation and regional specialization (Vaz et al., 2019). *nrg3b* transcripts were prominently observed in bilateral regions of the anterior forebrain and dorsal midbrain, including the optic tectum and dorsal diencephalon (Figure 4B). *gad1b* showed a broad expression pattern spanning the forebrain, midbrain, and hindbrain regions, with the highest signal in the optic tectum and ventral brain regions, which is consistent with the known pattern of GABAergic neurons (Silva et al., 2017; Liu et al., 2024^2^) (Figure 4C). In addition, *slc17a6a* showed a stronger signal in the dorsal forebrain and midbrain regions, identifying areas of excitatory neurons that spatially overlapped with *nrg3b* expression (Hiraki-Kajiyama et al., 2024) (Figure 4D), although definitive confirmation requires future double WISH analyses. This spatial correspondence aligns with observations in the mammalian brain, where Nrg3 has been shown to promote the formation of excitatory synapses on hippocampal interneurons in mice (Müller et al., 2018). Together, these findings suggest a putative role for NRG3 family members in the establishment and modulation of excitatory neuronal circuits in zebrafish.

### 3.4. Spatial expression of *nrg3b* in the adult zebrafish telencephalon

**Figure 5:**
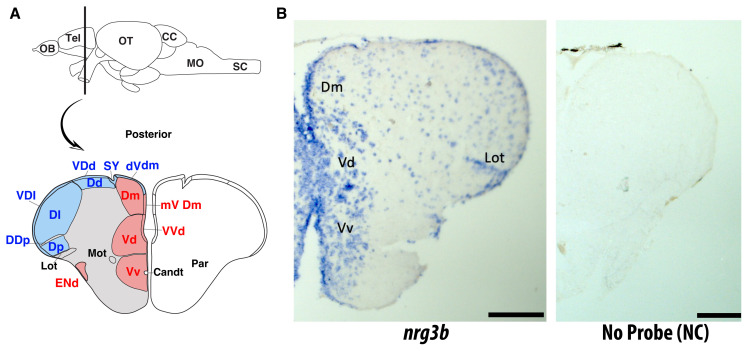
Spatial expression of *nrg3b* in the adult zebrafish telencephalon. A, The schematic view of the adult zebrafish forebrain shows the telencephalon’s major subregions in a coronal section. Areas corresponding to the dorsal (Dd, Dm, Dp) and ventral (Vd, Vv) domains of the telencephalon are indicated. Adapted conceptually from the telencephalic atlas published by (Diotel et al., 2015). B, ISH on coronal brain sections revealed expression of the *nrg3b* gene predominantly in the dorsomedial (Dm), ventral (Vd), and ventro-ventral (Vv) nuclei of the telencephalon. Scale bars: 50 μm. Stained tracts and nuclei overlapped with regions composed of rich populations of neurons and glial cells, which are involved in adult neurogenesis and the organization of neural circuits. The Lot was visible and served as an anatomical reference. No staining was observed in negative-control sections processed without probe (NC), confirming the specificity of the signal.

Chromogenic ISH on coronal sections of the adult zebrafish telencephalon was performed to identify the expression profiles of *nrg3b*. In the adult zebrafish brain, the telencephalon is divided into dorsal (pallium) and ventral (subpallium) regions, each with distinct neuronal populations (Mueller and Wullimann, 2009). The pallium (dorsomedial (Dm), Dd, Dl, and Dp) contains glutamatergic projection neurons and interneurons involved in sensory integration and learning (Ganz et al., 2010; Mueller et al., 2011), while the subpallium (Vd and Vv) harbors GABAergic and dopaminergic neurons as well as CSF-contacting cells, contributing to motor and olfactory processing (Rink and Wullimann, 2002). The lateral olfactory tract (Lot) links the olfactory bulb to telencephalic targets, mediating excitatory–inhibitory communication between dorsal and ventral domains (Miyasaka et al., 2014) (Figure 5A). Herein, the results showed distinct and localized signals in both the midline and peripheral regions of the telencephalon (Figure 5B). These expression domains are located within regions known to harbor diverse neuronal and glial populations, including radial glia, GABAergic interneurons, CSF-contacting neurons (CSF-CNs), and glutamatergic projection neurons; however, further cell-type-specific characterization will be required to determine which of these cell populations express *nrg3b*. Combined with embryonic data, these findings indicate that *nrg3b* is expressed in defined telencephalic regions during development and persists in the adult brain. Although chromogenic ISH does not allow precise resolution of cellular identity or relative expression levels, the observed expression pattern suggests a potential role for *nrg3b* in neural maturation, circuit formation, and sensory processing in the zebrafish brain.

## 4. Discussion

The integrated bioinformatics, in vitro, and in situ findings revealed that *nrg3b* is a developmentally regulated gene whose expression pattern and molecular context suggest a conserved role in synaptic signaling and neuronal organization in zebrafish.

The transcriptomic differences observed between heterozygous and maternal-zygotic mutant backgrounds most likely reflect differences in the effective dosage and functional integrity of Sspo, rather than the result of distinct, qualitative genotype-dependent mechanisms. Maternal-zygotic *sspo* alleles have been shown to permit early Reissner fiber assembly and near-normal embryonic morphology, followed by progressive fiber instability during larval stages, consistent with partial preservation of Sspo function (Troutwine et al., 2020). They lack maternal Sspo contribution and therefore experience reduced functional Sspo availability from the earliest stages of development, which may render the Reissner fiber more susceptible to instability at later developmental stages. In contrast, heterozygous animals retain maternal Sspo contribution and do not exhibit early embryonic axial defects, suggesting that maternal Sspo can partially support early CSF-dependent processes despite reduced zygotic dosage.

In this context, the PCA results herein show a clear temporal separation along the PC1 and maternal-zygotic embryos fall behind the heterozygotes at 5 dpf. This observation supports a developmental window during which the Sspo dosage becomes limiting for maintaining a stable CSF-dependent signaling environment. This distinction is consistent with the retention of maternal Sspo contribution in heterozygous embryos, in contrast to maternal-zygotic hypomorphic mutants, and therefore early-stage transcriptional sensitivity reflects graded Sspo function, rather than categorical, definitive effects of the mutants.

The mild divergence at 5 dpf is consistent with the role of Sspo in shaping CSF composition and signaling (Rose et al., 2020; Troutwine et al., 2020), suggesting that *nrg3b* expression responds to extracellular cues derived from CSF dynamics rather than direct genotypic control. Network analysis positioned *nrg3b* within a synaptic signaling module with genes involved in glutamatergic transmission and synaptic organization, including *nrxn2a*, *grid1b*, and *galntl6* (Gomez et al., 2021; Ung et al., 2024).

The strengthened coregulation among NRG paralogs and the broadened correlation of *nrg3b* with both excitatory and inhibitory markers in *sspo* mutants suggest that CSF perturbation can alter the transcriptional landscape in which Nrg3b operates. In mammals, NRG3 selectively binds to ErbB4, maintaining excitatory–inhibitory balance and cortical connectivity (Zhang et al., 1997; Müller et al., 2018). However, in the present study, transcriptional correlation analyses did not reveal a conserved coexpression relationship between *nrg3b* and *erbb* family members in zebrafish under *sspo* mutant backgrounds. This lack of transcriptional coupling suggests that, if present, Nrg3b–ErbB signaling in zebrafish may be regulated through mechanisms that are not reflected at the mRNA coexpression level, or that ErbB receptor regulation follows an independent transcriptional program. Therefore, resolving whether *nrg3b* participates in ErbB-mediated signaling in zebrafish will require targeted functional approaches, including cell-type-specific expression analyses and direct assessment of receptor activation.

The gradual increase in *nrg3b* expression from 1 dpf to 5 dpf suggests a role in early stages of neuronal differentiation and circuit formation. These results are consistent with a potential developmental involvement of Nrg3b in neural patterning, although the current data remain correlative and do not establish a causal role.

Embryonic WISH results revealed that *nrg3b* transcripts were detected in the forebrain, midbrain, and anterior spinal cord. When compared with the expression domains of *slc17a6a* (glutamatergic marker) and *gad1b* (GABAergic marker), *nrg3b* appeared more spatially aligned with *slc17a6a*-positive regions, while showing more limited correspondence with *gad1b*. These qualitative observations suggest that *nrg3b* is predominantly expressed in areas associated with excitatory neuronal populations. Moreover, in the adult brain, *nrg3b* expression persists in the Dm and ventral telencephalon regions (Vd and Vv). The Dm region has been proposed as the homolog of the mammalian pallial amygdala and is involved in fear conditioning and associative behaviors, reinforcing its functional overlap with limbic circuits in mammals (Lal et al., 2018; LeDoux, 2000; Marek et al., 2013). Molecular marker-based studies also indicate that subpallial regions such as Vd and Vv are counterparts of the basal ganglia and septal nuclei in the mammalian brain, linking these regions to motor, motivational, and signal integration functions (Ganz et al., 2015). Given the conserved organization of telencephalon subdivisions and gene expression patterns between zebrafish and mammals, the localization of *nrg3b* in these regions is consistent with potential roles beyond early development in the regulation and modulation of mature neural circuits.

In line with this anatomical and functional homology, *NRG3* has been implicated in human neuropsychiatric phenotypes through multiple genetic association studies, most prominently in relation to schizophrenia and symptom-related cognitive and emotional traits (Avramopoulos, 2018; Kao et al., 2010; Morar et al., 2011; Zeledón et al., 2015). Importantly, many of these functions are attributed to limbic and corticostriatal circuits, encompassing amygdala-related networks and basal ganglia pathways, which show strong anatomical and functional correspondence to the adult zebrafish telencephalic regions where *nrg3b* expression persists. In this context, the sustained expression of *nrg3b* in the adult zebrafish telencephalon supports the hypothesis that this gene may participate in conserved processes underlying emotional regulation, learning, and behavioral modulation in zebrafish, extending its relevance beyond early developmental stages (Ghaddar et al., 2021; Morizet et al., 2024).

## 5. Conclusion

Although this study combines transcriptomic analysis with anatomical validation, its scope remains exploratory. Combining bulk RNA-seq data, gene coexpression analysis, and histological assays provides a descriptive map of the *nrg3b* expression pattern but does not yet define its mechanistic function. The addition of the *sspo* gene as a candidate upstream transcriptomic regulator links Nrg3b to CSF-dependent regulatory pathways.

In summary, this study provides qualitative and descriptive baseline that *nrg3b* is involved in neuronal developmental signaling processes in zebrafish. Its developmental increase in expression, spatial restriction to excitatory regions, and persistence of expression in mature telencephalon nuclei make this gene a strong candidate for mediating synaptic organization and maintenance of neural circuits. Future studies will need to use quantitative imaging, cell-type-specific validation, and genetic manipulations to identify the precise cell populations expressing *nrg3b* and to determine its functional role in neuronal differentiation and communication. This research forms a foundation for a more detailed investigation of the Nrg3b signaling and its role in vertebrate brain development by establishing a descriptive foundation and presenting testable hypotheses.

## Supplementary Data

Figure S Spatiotemporal expression pattern of the *nrg3b* in zebrafish neuronal cells based on single-cell RNA sequencing data from the Daniocell database^3^. In the Daniocell single-cell RNA sequencing dataset *nrg3b* gene expression is mainly observed in the neuronal population of zebrafish embryos. Left, UMAP of 165,054 neuronal cells (33.71% of total, n = 489,686) colored according to the developmental stage (5–120 h postfertilization (hpf). Each dot represents a single cell positioned according to transcriptional similarity. The color spectrum from purple/orange to green/blue indicates a gradual transition from early progenitor cells to late differentiated neuronal stages. Right, UMAP of the same cell set colored according to the logarithmic expression level of the *nrg3b* gene (log_2_). Warmer colors (yellow–red) indicate higher expression, mainly concentrated in the neural crests of embryos at 5 dpf ≈ 120 hpf. This restricted expression pattern reflects the temporal and spatial regulation of the *nrg3b* gene during neural differentiation. Data were obtained from the Daniocell zebrafish single-cell atlas.

**Table S1:** Primer sequences used in WISH and ISH.

Primer name	Primer sequence (5′-3′)
Nrg3b-F	5′TCCGACCACCACTAGCACGAC-3′
Nrg3b T7-R	5′- ATATATTAATACGACTCACTATAGGAGACATGAGACCAGAGGCGTT-3′
Lbx1a-F	5′-ACACTGACTTCCAGCCCCTTGAAGG-3′
Lbx1a T7-R	5′- ATATATTAATACGACTCACTATAGGACACTGACTTCCAGCC-3′
Gad1b-F	5′-AGATGCGCTCTTCTCACCTG-3′
Gad1b T7-R	5′- ATATATTAATACGACTCACTATAGGAGATGTCCACATGTCTGCCG-3′
Slc17a6a-F	5′-CTCAGGCTGGTCTTCCGTCT-3′
Slc17a6a T7-R	5′- ATATATTAATACGACTCACTATAGGCGTGGCGCAATGTCCAAG-3′

**Table S2:** Primer sequences used in the RT-qPCR.

Primer name	Primer sequence (5′-3′)
Nrg3b_RT-PCR forward	5′-AGGGGTGAAAACCACGCAGTA-3′
Nrg3b_RT-PCR reverse	5′-TTCGTCCCCCGTGAATGAAG-3′
Rpl13a_RT-PCR forward	5′-CCCTCCACCTTATGACAAGAGA-3′
Rpl13a_RT-PCR reverse	5′-CGTCCAAGCAGGGCAAATTT-3′

## Figures and Tables

**Table:** Summary of the RNA-seq samples used for transcriptomic analysis, including heterozygous (*sspo_hsc105/stl297_*) and maternal-zygotic (*sspo_stl297/stl297_*) mutant zebrafish at 5, 15, and 30 dpf. Each condition at each time point includes 3 biological replicates.

Time (dpf)	Condition	Sample IDs
5	Heterozygous	5dpf_het_17, 5dpf_het_18, 5dpf_het_19
	Maternal-zygotic	5dpf_mat_20, 5dpf_mat_21, 5dpf_mat_22

15	Heterozygous	15dpf_het_23, 15dpf_het_24, 15dpf_het_25
	Maternal-zygotic	15dpf_mat_26, 15dpf_mat_27, 15dpf_mat_28

30	Heterozygous	30dpf_het_29, 30dpf_het_30, 30dpf_het_31
	Maternal-zygotic	30dpf_mat_32, 30dpf_mat_33, 30dpf_mat_34

## Footnotes

1 Daniocell (n.d.). Daniocell zebrafish single-cell atlas [online]. Website: https://eur03.safelinks.protection.outlook.com/?url=https%3A%2F%2Fdaniocell.nichd.nih.gov%2F&data=05%7C02%7Cnuray.erdogan%40khas.edu.tr%7C901c008026764bc9140e08de57540a2a%7Cf2ea0fb27d344ebf9e5364ec41288dbb%7C0%7C0%7C639044215626587444%7CUnknown%7CTWFpbGZsb3d8eyJFbXB0eU1hcGkiOnRydWUsIlYiOiIwLjAuMDAwMCIsIlAiOiJXaW4zMiIsIkFOIjoiTWFpbCIsIldUIjoyfQ%3D%3D%7C0%7C%7C%7C&sdata=FrANhyyj%2B%2F%2B0aDIv85BV5w%2FNBLCsveSBUDjaV8bubj0%3D&reserved=0 [accessed 19 January 2025]

2 Liu Y, Chen Y, Duffy CR, VanLeuven AJ, Byers JB et al. (2024). Decreased GABA levels during development result in increased connectivity in the larval zebrafish tectum [preprint]. bioRxiv. Website: https://www.biorxiv.org/content/10.1101/2024.09.11.612511v1 [accessed 30 March 2025].

3 Daniocell (n.d.). Daniocell zebrafish single-cell atlas [online]. Website: https://eur03.safelinks.protection.outlook.com/?url=https%3A%2F%2Fdaniocell.nichd.nih.gov%2F&data=05%7C02%7Cnuray.erdogan%40khas.edu.tr%7C901c008026764bc9140e08de57540a2a%7Cf2ea0fb27d344ebf9e5364ec41288dbb%7C0%7C0%7C639044215626587444%7CUnknown%7CTWFpbGZsb3d8eyJFbXB0eU1hcGkiOnRydWUsIlYiOiIwLjAuMDAwMCIsIlAiOiJXaW4zMiIsIkFOIjoiTWFpbCIsIldUIjoyfQ%3D%3D%7C0%7C%7C%7C&sdata=FrANhyyj%2B%2F%2B0aDIv85BV5w%2FNBLCsveSBUDjaV8bubj0%3D&reserved=0 [accessed 19 January 2025]
