# Supplementary figures and images for: Transcriptomic identification and developmental mapping of nrg3b expression in zebrafish
Source: Turk J Biol. 2025 Dec 29;50(1):81–93. doi: 10.55730/1300-0152.2791 (PMC12978766; doi:10.55730/1300-0152.2791)

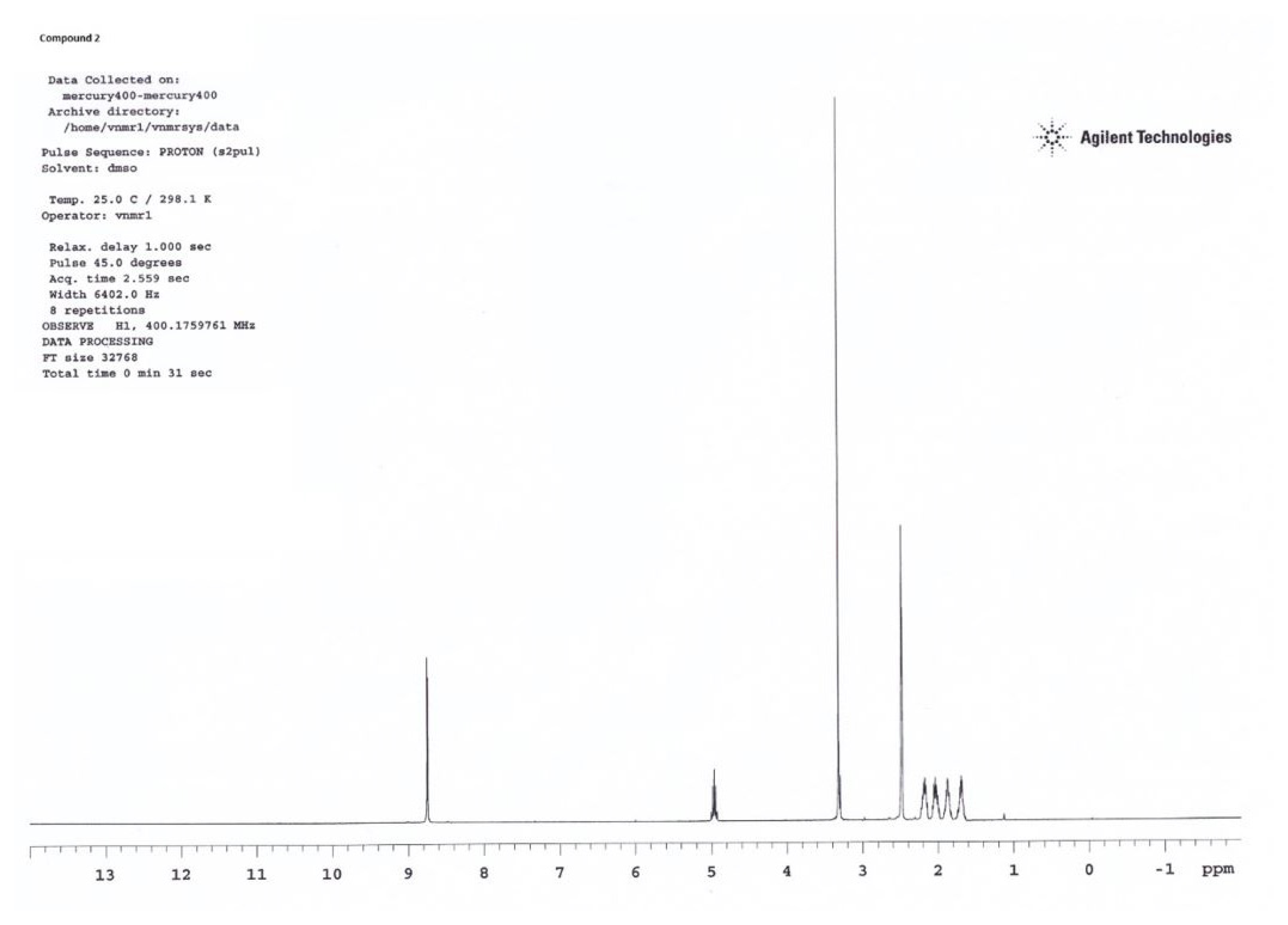

Supplement: Figure S — Spatiotemporal expression pattern of the nrg3b in zebrafish neuronal cells based on single-cell RNA sequencing data from the Daniocell database3. In the Daniocell single-cell RNA sequencing dataset nrg3b gene expression is mainly observed in the neuronal population of zebrafish embryos. Left, UMAP of 165,054 neuronal cells (33.71% of total, n = 489,686) colored according to the developmental stage (5–120 h postfertilization (hpf). Each dot represents a single cell positioned according to transcriptional similarity. The color spectrum from purple/orange to green/blue indicates a gradual transition from early progenitor cells to late differentiated neuronal stages. Right, UMAP of the same cell set colored according to the logarithmic expression level of the nrg3b gene (log2). Warmer colors (yellow–red) indicate higher expression, mainly concentrated in the neural crests of embryos at 5 dpf ≈ 120 hpf. This restricted expression pattern reflects the temporal and spatial regulation of the nrg3b gene during neural differentiation. Data were obtained from the Daniocell zebrafish single-cell atlas. [file tjb-50-01-29s1.tif]
